# Genome-Wide Identification and Expression Analysis of the HSP90 Gene Family in Relation to Developmental and Abiotic Stress in Ginger (*Zingiber officinale* Roscoe)

**DOI:** 10.3390/plants14111660

**Published:** 2025-05-29

**Authors:** Daoyan Xiao, Yajun Jiang, Zhaofei Wang, Xingyue Li, Hui Li, Shihao Tang, Jiling Zhang, Maoqin Xia, Meixia Zhang, Xingfeng Deng, Hong-Lei Li, Huanfang Liu

**Affiliations:** 1 Chongqing Engineering Research Center for Horticultural Plant, College of Smart Agriculture, Chongqing University of Arts and Sciences, Chongqing 402160, China; xdy19110910919@163.com (D.X.); jiangyajun228@163.com (Y.J.); wangzhaofei2025@163.com (Z.W.); lixingyue015@163.com (X.L.); lihui145327@163.com (H.L.); tangshihao00@163.com (S.T.); 13114062660@163.com (J.Z.); xiamq@cqwu.edu.cn (M.X.); 20110067@cqwu.edu.cn (M.Z.); 2 State Key Laboratory of Plant Diversity and Specialty Crops, Guangdong Provincial Key Laboratory of Applied Botany, Guangdong Provincial Key Laboratory of Digital Botanical Garden, South China Botanical Garden, Chinese Academy of Sciences, Guangzhou 510650, China; 3 Industrial Development Service Center of Baizi, Tongnan, Chongqing 402160, China; 13883487206@163.com; 4 Chongqing Key Laboratory for Germplasm Innovation of Special Aromatic Spice Plants, College of Smart, Agriculture, Chongqing University of Arts and Sciences, Chongqing 402160, China

**Keywords:** ginger, *HSP90* genes, expression patterns, development, stress responses

## Abstract

Ginger (*Zingiber officinale* Roscoe), valued both for its medicinal and culinary uses, can be adversely affected by abiotic stresses such as high temperature and drought, which can impact its growth and development. The HSP90 gene family has been recognized as a crucial element for enhancing heat and drought resistance in plants. Nevertheless, no studies have yet reported on the HSP90 gene family in ginger. This study investigates the HSP90 gene family in ginger and its crucial role in the plant’s responses to abiotic stresses. A total of 11 ZoHSP90 members were identified in the ginger genome, and these genes were unevenly distributed across five chromosomes. Bioinformatics analyses revealed that the HSP90 proteins in ginger vary in size, ranging from 306 to 886 amino acids. These proteins are predominantly located in the cytoplasm, endoplasmic reticulum, and mitochondria. Notably, ten conserved motifs were identified, with variations in motif distribution correlating with phylogenetic relationships among the genes. Furthermore, the gene structure analysis indicated differences in exon numbers, which may reflect specialized regulatory mechanisms and functional differentiation among the *ZoHSP90* genes. Cis-acting elements within the promoter regions of the *ZoHSP90* genes were identified, and their involvement in stress responses and hormonal signaling pathways was revealed. These elements are critical for regulating gene expression patterns in response to environmental stimuli, such as methyl jasmonate, salicylic acid, and abscisic acid. The presence of these elements indicates that *ZoHSP90* genes play significant regulatory roles in plant adaptation to environmental changes. Expression profiling of the *ZoHSP90* genes under various abiotic stress conditions demonstrated tissue specificity and dynamic regulation. Different *ZoHSP90* genes exhibited distinct expression patterns in response to low-temperature, drought, high-temperature, and salt stresses. This suggests that the HSP90 gene family in ginger possesses both conserved functions and species-specific adaptations to optimize stress responses. Overall, this research provides valuable insights into the molecular functions of the HSP90 gene family in ginger and lays the groundwork for future studies aimed at enhancing crop resilience through genetic engineering. The findings contribute to a deeper understanding of plant adaptability to environmental stressors, which is crucial for improving agricultural productivity in the face of climate change.

## 1. Introduction

Research indicates that globally, crop yields, including those of heat-sensitive crops, are vulnerable to increasing climate variability and heat stress [1,2,3]. Extreme heat events, under future climate scenarios, could lead to significant reductions in crop productivity, with some regions projected to experience yield declines of up to 30% for major crops [4]. In recent years, rising global temperatures and the increasing frequency of extreme weather events, such as heatwaves, have severely impacted global agriculture [5,6]. These temperature extremes have caused significant damage to crops, including ginger, a key agricultural product that has become an important pillar of the economy in several regions of China [7]. Ginger, valued both for its medicinal and culinary uses, requires specific environmental conditions for optimal growth. During its active growth phase, ginger thrives at temperatures around 27.5 °C; however, when temperatures exceed 35 °C, photosynthesis is impaired, and plant growth is significantly reduced [8]. Climate change-associated temperature increases and prolonged drought directly threaten ginger production through yield reduction and rhizome quality deterioration. These threats consequently jeopardize both food security and agricultural livelihoods. As the need for climate-resilient crops grows, identifying genes responsible for heat tolerance in ginger and employing molecular breeding techniques will be essential for ensuring the sustainability of ginger production under increasingly extreme conditions.

The HSP90 gene family is a highly conserved group of molecular chaperones that is widely present in both animals and plants [9,10,11,12]. The HSP90 gene family is widely distributed in cells, exhibiting significant structural and evolutionary characteristics. The HSP90 gene family has been extensively studied in plants such as Arabidopsis, oats, pumpkins, and cucumbers. The expansion of these genes primarily occurs through duplication, demonstrating diversity and conservation in gene structure and conserved motifs. Based on their evolutionary relationships and functions, the HSP90 gene family is classified into different groups [11,13,14,15]. In oats, the HSP90 gene family consists of 20 genes, exhibiting structural conservation, especially in the branches of the phylogenetic tree [16]. In cabbage, the *HSP90* genes are classified into five groups, showing collinearity with Arabidopsis and other plants, indicating the conservation of their gene structures. Most *HSP90* genes contain few introns and are found in the genome as tandem repeats or segmental duplications. In Arabidopsis, cytosolic *HSP90* genes have fewer introns, whereas organellar *HSP90* genes contain more introns [17].

HSP90 is widely present in both prokaryotic and eukaryotic organisms. It is induced by environmental stimuli, functions as a molecular chaperone, and participates in regulating plant growth, development, and stress responses. In recent years, with the establishment of numerous plant gene databases, HSP90 has been identified in an increasing number of species. The 90 kDa heat shock protein (HSP90), a member of the heat shock protein family, plays a crucial role in refolding misfolded proteins accumulated during various stress responses and facilitates the degradation of mutated proteins. In lily, heterologous expression of the *LrHSP17.2* transgene enhances the activities of antioxidant enzymes, including peroxidase and superoxide dismutase (SOD), thereby facilitating the scavenging of excess reactive oxygen species (ROS). This activity contributes to the preservation of membrane structural integrity and the regulation of genes involved in antioxidant defense pathways under abiotic stress conditions [18]. In perennial ryegrass, all *HSP90* genes are highly expressed under heat stress [19]. In cucumbers, six *HSP90* genes have been identified, exhibiting distinct expression patterns and stress responses across various tissues. Notably, *Csa1G569270* and *Csa1G569290* show differential expression under both biotic and abiotic stress conditions [11]. In Arabidopsis, overexpression of five *HSP90* genes increased proline accumulation, thereby enhancing resistance to abiotic stress such as high temperature and drought [20]. In tobacco, heat stress induces the expression of 21 HSP90 family members, with all showing similar response patterns under drought or extreme temperature stress [21]. In apples, four *HSP90* members are involved in the response to high-temperature stress [22]. In conclusion, the HSP90 gene family plays a crucial role in plant responses to high temperatures and drought stress, and overexpression of HSP90 enhances crop heat and drought resistance.

Currently, no studies have reported on the HSP90 gene family in ginger. The present study identified members of the HSP90 gene family using the ginger genome assembled by our team [23]. We analyzed their physicochemical properties, gene structures, conserved motifs, and evolutionary characteristics. Additionally, we clarified the distribution characteristics of the HSP90 gene family within the genome and identified the cis-acting elements in their promoters. Using qRT-PCR technology, we investigated the expression levels of these genes under high-temperature and strong light stress. This research provides a foundational understanding for further exploring the functions of HSP90 in ginger and offers theoretical guidance for the development of high-temperature and strong light cultivation techniques and molecular breeding for heat resistance in ginger.

## 2. Results

### 2.1. Identification and Physicochemical Properties of Ginger HSP90 Genes

Through the application of the hmmsearch program and screening against the Pfam database, a total of 11 members of the HSP90 gene family were identified after redundancy removal and were designated as *ZoHSP90.1* to *ZoHSP90.11.* The physicochemical properties of these 11 *HSP90* sequences were analyzed, and subcellular localization predictions were conducted (Table 1). The amino acid lengths of the HSP90 family members ranged from 306 to 886 amino acids, with molecular weights varying from 34.04 to 101.6 kDa. Notably, *ZoHSP90.7* exhibited the longest sequence and the highest molecular weight, while the other eight members had shorter sequences and lower molecular weights. The theoretical isoelectric points (pI) of the HSP90 proteins ranged from 4.51 to 5.47, indicating that they are predominantly acidic. The instability index varied from 31.33 to 42.07, with *ZoHSP90.1* having an index of 42.07, suggesting it may be less stable, whereas the other members displayed better stability. Subcellular localization predictions suggest that four members of the ginger HSP90 family are localized to the endoplasmic reticulum, five to the cytoplasm, and two to both the endoplasmic reticulum and mitochondria.

**Figure 1:**
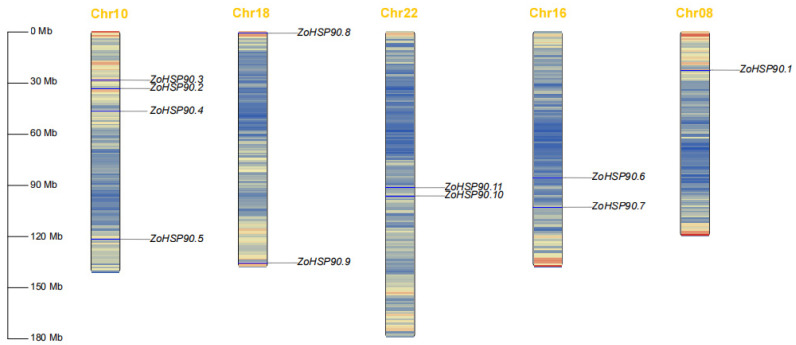
Chromosomal localization of HSP90 gene family members in ginger.

According to the genetic linkage map (Figure 1), the 11 HSP90 gene family members are distributed across five chromosomes. Specifically, *ZoHSP90.3*, *ZoHSP90.2*, *ZoHSP90.4*, and *ZoHSP90.5* are located on chromosome 10, *ZoHSP90.8* and *ZoHSP90.9* are found on chromosome 18, *ZoHSP90.10* and *ZoHSP90.11* are situated on chromosome 22, *ZoHSP90.6* and *ZoHSP90.7* are located on chromosome 16, and *ZoHSP90.1* is found on chromosome 8. This distribution suggests a non-random arrangement of *HSP90* genes within the ginger genome, which may have implications for their functional interactions and evolutionary history.

### 2.2. Synteny Analysis of the HSP90 Gene Family in Ginger

**Figure 2:**
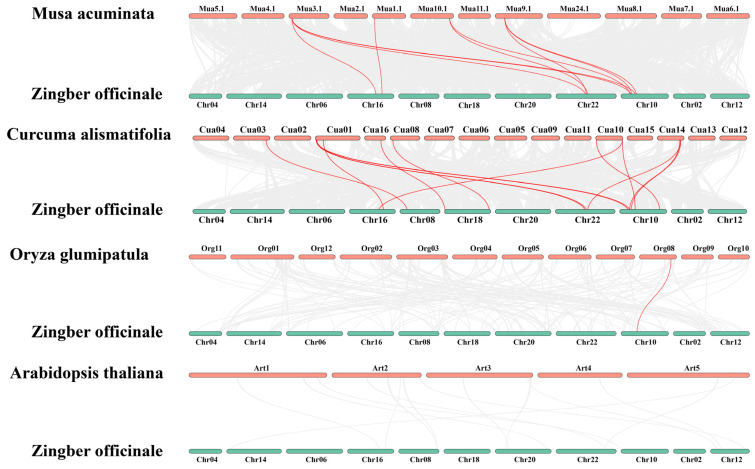
Interspecies synteny analysis of the HSP90 genes among *Musa acuminata* (wild banana), *Zingiber zerumbet* (shampoo ginger), *Oryza sativa* (rice), *Arabidopsis thaliana* (Arabidopsis), and *Zingiber officinale* (ginger).

**Figure 3:**
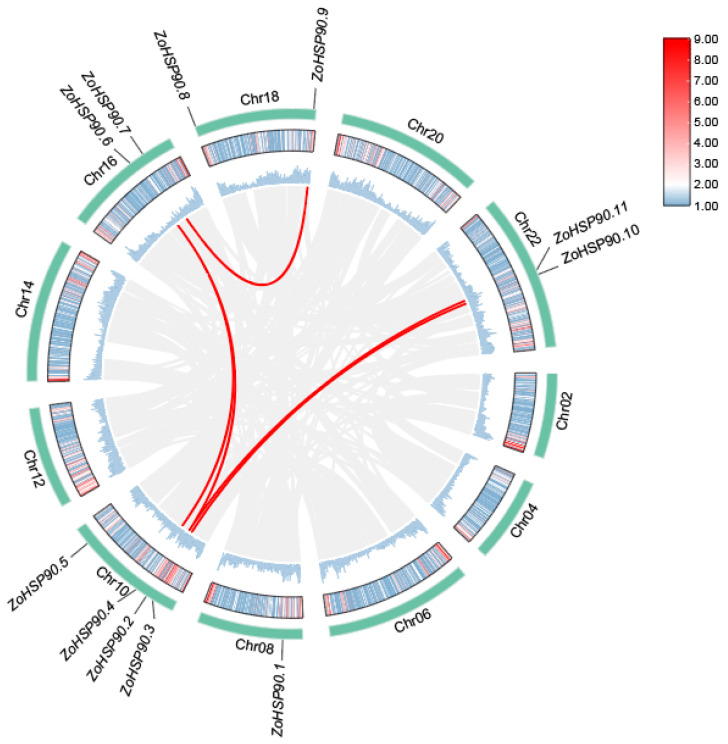
Intraspecies synteny analysis of the HSP90 gene family in ginger, highlighting homologous relationships among gene members.

To further investigate the homologous relationships of the HSP90 gene family, a multi-species synteny analysis was conducted (Figure 2), selecting HSP90 family members from *Musa acuminata* (wild banana), *Zingiber zerumbet* (shampoo ginger), *Oryza sativa* (rice), and *Arabidopsis thaliana* (Arabidopsis) for comparison, alongside an intraspecies synteny analysis of the ginger HSP90 family (Figure 3). The interspecies synteny analysis revealed that four HSP90 members in *Musa acuminata* are directly homologous to nine HSP90 members in ginger, forming four pairs of orthologous relationships. Additionally, nine HSP90 members in *Zingiber zerumbet* are directly homologous to fourteen HSP90 members in ginger, resulting in six pairs of orthologous relationships. Furthermore, one HSP90 member in *Oryza sativa* is directly homologous to one HSP90 member in ginger, forming one pair of orthologous relationships. However, no direct homologous relationships were found between the HSP90 members of *Arabidopsis thaliana* and those in ginger. Five pairs of segmental duplication genes were detected by intraspecific collinearity analysis, involving eight Zo *HSP90* genes. Among them, *HSP90.7* is homologous to *HSP90.9*, and *HSP90.6* is homologous to *HSP90.4* and *HSP90.2.* In addition, *HSP90.11* is homologous to *HSP90.2*, and *HSP90.10* is homologous to *HSP90.3*.

The Ka/Ks ratio can be used to assess the selection process history of coding sequences [24]. Table 2 presents the evolutionary analysis results of five paralogous pairs of *HSP90* genes. The Ka/Ks ratios for each gene pair are significantly less than 1 (ranging from 0.02 to 0.11), indicating strong purifying selection during evolution and suggesting that their functions are highly conserved. The duplication times range from 6.8 to 17.0 million years ago (Mya), with the earliest duplication event in *ZoHSP90.6*-*ZoHSP90.2* (17.0 Mya) and the most recent in *ZoHSP90.10*-*ZoHSP90.3* (6.8 Mya). The Ks values (synonymous substitution rates) range from 0.41 to 1.02, further supporting that these duplication events occurred relatively recently in evolutionary terms. Overall, these gene duplication events likely took place at different time points, but all duplicated genes are under functional constraints, implying their biological importance.

### 2.3. Phylogenetic Analysis of the HSP90 Gene Family

To further understand the phylogenetic relationships and functional characteristics of the HSP90 gene family in ginger, HSP90 protein sequences were downloaded from the NCBI database, comprising 7 sequences from *Arabidopsis thaliana*, 11 from *Solanum tuberosum* (potato) [25], and 9 from *Oryza sativa* (rice) [26]. These were aligned with the 11 identified HSP90 protein sequences from ginger to construct a phylogenetic tree. The phylogenetic analysis revealed the division of the HSP90 gene family into four subfamilies (Groups A-D). Specifically, *AtHSP90.7* and *ZoHSP90.4* formed Group A, *OsHSP90.7* and *OsHSP90.3* constituted Group B, *StHSP90.7* and *OsHSP90.1* were classified into Group C, and *AtHSP90.5* and *ZoHSP90.3* made up Group D. Cluster analysis indicated that the *ZoHSP90* genes are unevenly distributed across two subfamilies. Notably, a high level of homology was observed between the *HSP90* genes of ginger and Arabidopsis, suggesting that the orthologous genes may share similar functions.

### 2.4. Analysis of Conserved Motifs and Gene Structure of HSP90 in Ginger

**Figure 4:**
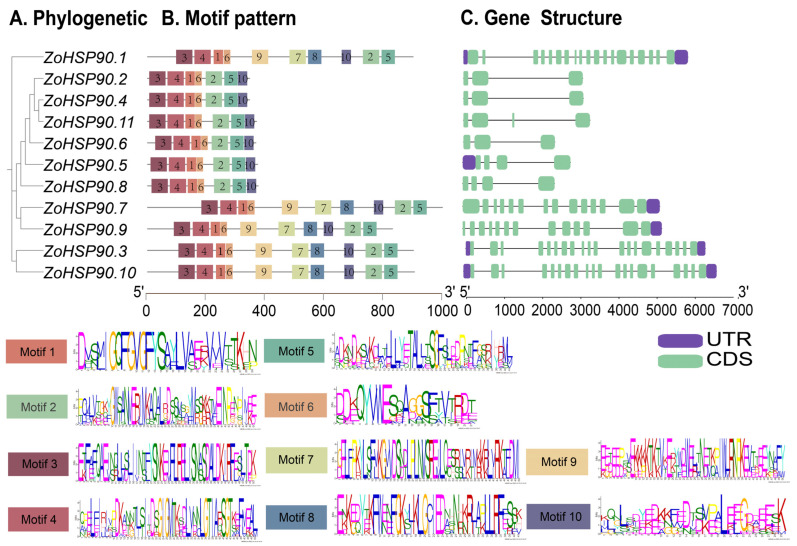
Conserved motifs and gene structures of the HSP90 gene family in ginger. (**A**) Maximum likelihood phylogenetic tree of ZoHSP90 proteins based on full-length amino acid sequences. (**B**) Distribution of ten conserved motifs identified by MEME analysis, with motif composition indicated by colored boxes (key in panel legend). (**C**) Exon-intron organization of *ZoHSP90* genes. Green rectangles represent coding sequences (CDS), purple rectangles denote untranslated regions (UTRs), and connecting lines indicate introns.

A total of 10 conserved motifs were identified in the HSP90 proteins of ginger (Figure 4). Different letters represent different amino acids. The higher the letter height, the greater the conservation of the amino acid at that position. Among these, *ZoHSP90.1*, *ZoHSP90.7*, *ZoHSP90.9*, *ZoHSP90.3*, and *ZoHSP90.10* each contained all 10 conserved motifs. In contrast, the distribution of motifs in *ZoHSP90.2*, *ZoHSP90.4*, *ZoHSP90.11*, *ZoHSP90.6*, *ZoHSP90.5*, and *ZoHSP90.8* was more uniform, with each of these proteins exhibiting seven conserved motifs. Comparative analysis revealed that genes with closer phylogenetic relationships exhibited greater similarity in both the number and position of motifs, indicating a high degree of conservation. This suggests that their structural domains and functional units are fundamentally similar. In terms of gene structure, it was found that *ZoHSP90.1*, *ZoHSP90.3*, and *ZoHSP90.10* possess a higher number of exons, with counts of 19, 20, and 20, respectively. Conversely, *ZoHSP90.2*, *ZoHSP90.4*, *ZoHSP90.11*, *ZoHSP90.6*, *ZoHSP90.5*, and *ZoHSP90.8* have fewer exons, with counts of 3, 3, 4, 3, 4, and 4, respectively. This variation in exon number may reflect differences in the functional complexity and regulatory mechanisms of these *ZoHSP90* genes in ginger.

### 2.5. Cis-Acting Elements in the Promoters of HSP90 Genes in Ginger

**Figure 5:**
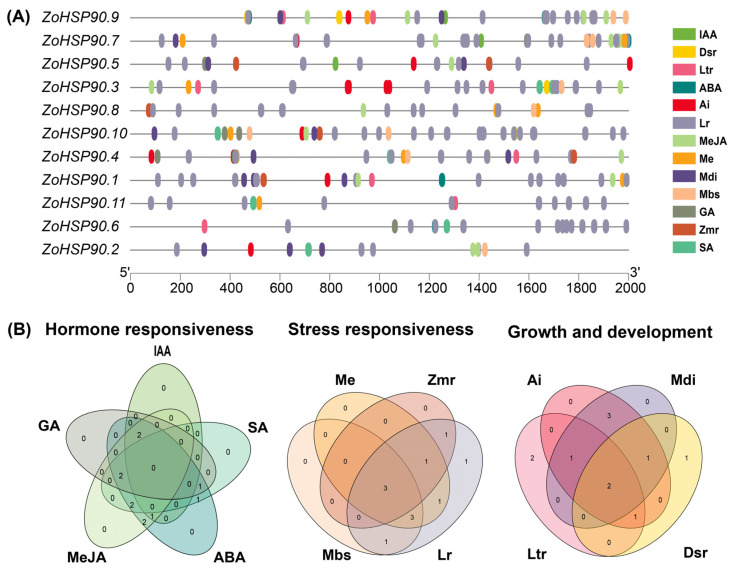
Cis-Regulatory Element Profiling of Ginger HSP90 Genes. (**A**) Distribution of cis-acting elements in *ZoHSP90* promoters. IAA: auxin-responsive element; GA: gibberellin-responsive element; MeJA: MeJA-responsiveness; ABA: abscisic acid responsiveness; SA: salicylic acid responsiveness; Me: meristem expression; Zmr: zein metabolism regulation; Mbs: MYBHv1 binding site; Lr: light responsiveness; Ai: anaerobic induction; Mdi: (MYB) drought-inducibility; Dsr: defense and stress responsiveness; Ltr: low-temperature responsiveness. (**B**) Overlap analysis of stress-responsive cis-element classes among *ZoHSP90* promoters.

A cis-acting element analysis was conducted on the promoters of the HSP90 gene family in ginger (Figure 5). In addition to the core elements in the promoter regions, the analysis focused on the distribution of elements related to hormone regulation and abiotic stress responses. The analysis revealed a substantial presence of various cis-acting elements associated with light response, stress response, and hormone regulation across the promoters of the 11 *HSP90* genes in ginger. Specifically, the following elements were identified: 144 light response elements, 36 abscisic acid (ABA) response elements, 40 methyl jasmonate response elements, 7 gibberellin response elements, 7 salicylic acid response elements, and 8 low-temperature response elements, which may play a significant role in temperature regulation. Additionally, the promoters contained 6 defense and stress response elements, 13 hypoxia response elements, and 13 drought response elements. To facilitate the understanding of the relationships among these elements, the hormone regulation, abiotic stress response, and growth development-related elements were analyzed and categorized. The results were visualized using a Venn diagram, highlighting the overlaps and unique elements among the different categories. This analysis underscores the potential regulatory mechanisms that the HSP90 gene family may employ in response to environmental stimuli and hormonal signals, contributing to the adaptability of ginger to various stress conditions.

### 2.6. Analysis of Gene Expression Characteristics of Ginger HSP90

**Figure 6:**
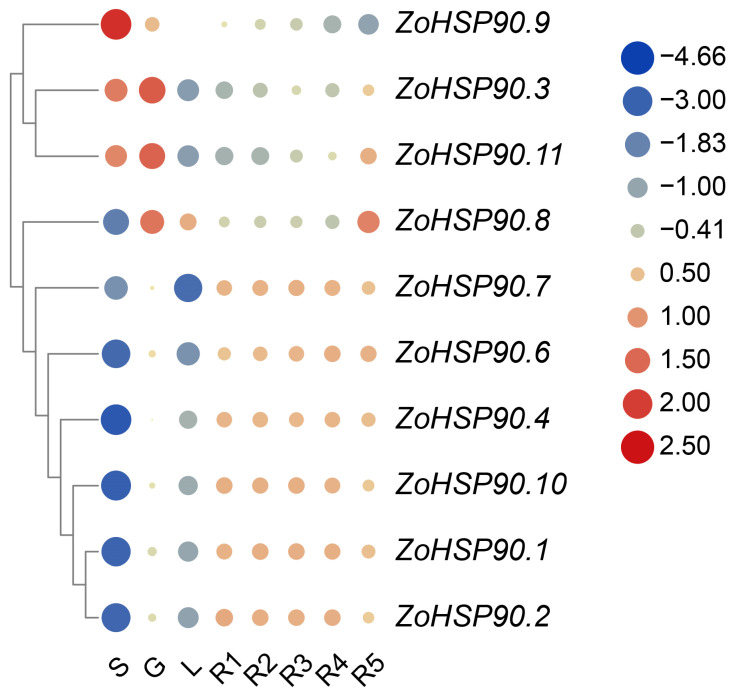
Gene expression heatmap of the ginger HSP90 gene family across different organs. Abbreviations: S denotes stem; G denotes root; L denotes leaves; R1 denotes primary rhizome buds; R2 denotes secondary rhizome buds; R3 denotes tertiary rhizome buds; R4 denotes fourth rhizome buds; R5 denotes fifth rhizome buds. The right column represents gene expression levels, with red coloring indicating upregulation, while blue denotes downregulation.

Utilizing the gene expression profile data of ginger *HSP90* genes, a gene expression heatmap was constructed to analyze the gene expression characteristics (Figure 6). The heatmap revealed that *HSP90* genes exhibit expression in the roots, stems, leaves, and rhizomes of ginger. Specifically, *ZoHSP90.9* exhibited the highest expression level in the stems, while *ZoHSP90.3* and *ZoHSP90.11* showed the highest expression levels in the roots. *ZoHSP90.8* displayed relatively high expression levels in both the roots and fifth-level rhizomes. On the other hand, *ZoHSP90.1*, *ZoHSP90.2*, *ZoHSP90.4*, *ZoHSP90.6*, *ZoHSP90.7*, and *ZoHSP90.10* exhibited lower expression levels in the stems, indicating potential roles of *ZoHSP90.9* in stem growth and development, and *ZoHSP90.3* and *ZoHSP90.11* in regulating root growth and development.

### 2.7. Expression Analysis of Ginger HSP90 Genes in Response to Low-Temperature, Drought, High-Temperature, and Salt Stress

**Figure 7:**
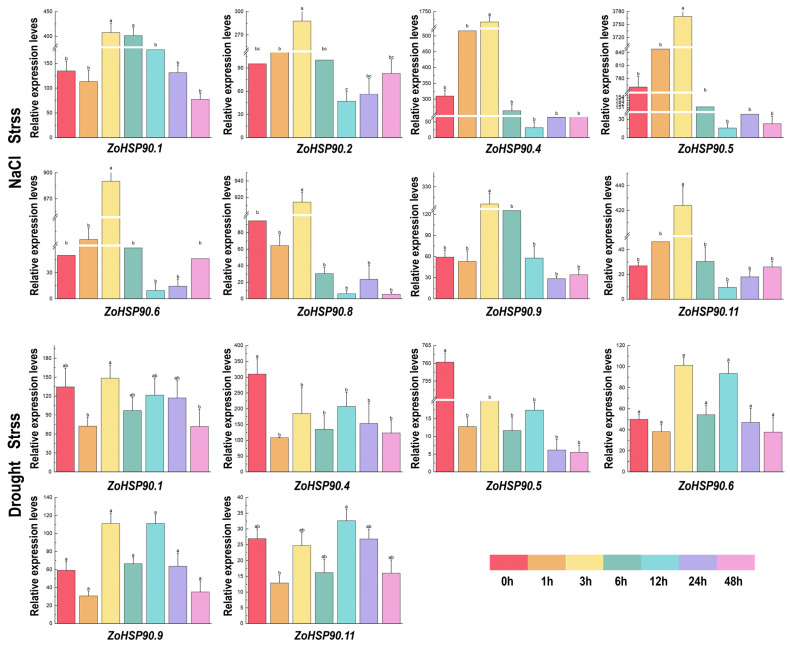
Expression patterns of *Zingiber officinale HSP90* genes under drought and NaCl stress. Different lowercase letters above the columns indicate significant differences among treatments at *p* < 0.05.

Based on RNA-seq data of stress-responsive genes, the expression patterns of ginger *HSP90* genes under low-temperature, drought, high-temperature, and salt stress conditions exhibited significant differences (Figure 7). In low temperatures, *ZoHSP90.4* reached its peak expression at 6 h, then gradually decreased, reaching its lowest expression level at 48 h. Additionally, *ZoHSP90.1* displayed its maximum expression at 1 h. Under high-temperature conditions, expression levels of *ZoHSP90.1* and *ZoHSP90.9* peaked after 12 h of stress, then gradually decreased. Under drought stress conditions, *ZoHSP90.5* exhibited minimal expression at 1 h, whereas *ZoHSP90.6* and *ZoHSP90.9* displayed a similar expression trend with higher levels at 3 and 12 h. Under salt stress conditions, *ZoHSP90.4*, *ZoHSP90.5, ZoHSP90.6*, *ZoHSP90.8*, and *ZoHSP90.11* showed almost no expression after 3 h. This analysis indicates that different *HSP90* genes in ginger respond distinctly to various environmental stressors, highlighting their potential roles in stress tolerance mechanisms.

### 2.8. QRT-PCR Analysis of Ginger HSP90 Gene Family Members

**Figure 8:**
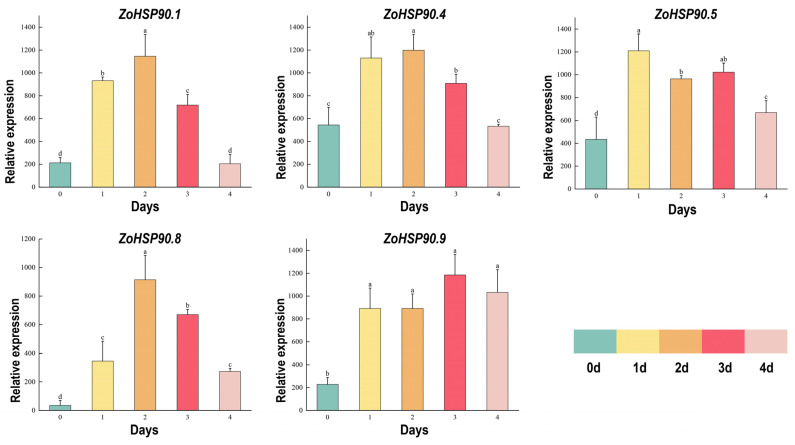
QRT-PCR analysis of ginger *HSP90* gene expression under high-temperature and strong light stress. Data were normalized to the *TUB-2* gene, with vertical bars representing the standard deviation. Different lowercase letters above the bars indicate significant differences between treatments (*p* < 0.05).

The expression levels of ginger HSP90 genes under high temperatures and strong light stress were assessed using qRT-PCR (Figure 8). The results demonstrated that all 11 ginger *HSP90* genes showed an upregulation pattern following high-temperature stress. Specifically, *ZoHSP90.1*, *ZoHSP90.4*, and *ZoHSP90.8* exhibited similar expression profiles, with an initial increase followed by a decrease, reaching their peak expression levels at 2 days post-stress. *ZoHSP90.1* showed a 4.5-fold increase relative to the control after 1 day, while *ZoHSP90.4* exhibited a 2-fold increase. In contrast, *ZoHSP90.5* displayed a distinct expression pattern, peaking on day 1, downregulating to 1.3 times the control on day 2, then gradually increasing again by day 3, and further rising on day 4. *ZoHSP90.9* showed a significant increase to 4.25 times the control between days 1 and 2, followed by a further upregulation to 1.4 times on day 3. This qRT-PCR analysis offers valuable insights into the dynamic expression patterns of the *HSP90* genes in ginger under high-temperature and strong light stress conditions, underscoring their potential roles in stress response mechanisms.

## 3. Discussion

In recent years, the increasing frequency of extreme weather events has significantly impacted crop growth, development, and yield due to abiotic stresses such as high temperatures, drought, and soil salinization [27]. The HSP90 family plays a vital role in plant development, responses to stressful environments, and disease resistance [28]. By regulating the stability and activity of target proteins, HSP90 helps plants maintain physiological balance under stress conditions, thereby promoting growth and development. Moreover, the expression of *HSP90* is modulated by various environmental factors, including temperature, drought, and salinity. This indicates that *HSP90* has a significant adaptive role in helping plants cope with abiotic stresses [29,30,31]. Understanding the functions of the HSP90 gene family under different environmental conditions is essential for enhancing crop resilience and optimizing agricultural production.

This study identified a total of 11 *HSP90* genes in ginger. Compared to the identification of six *HSP90* genes in Arabidopsis [15], 10 in popla [13], and 7 in tomato [21], ginger has the largest number of identified *HSP90* genes. Gene duplication events are a crucial evolutionary mechanism driving gene expansion in plants [32]. In this study, we did not identify any tandemly duplicated genes, but we did detect five pairs of segmentally duplicated genes. The presence of segmental duplications on different chromosomes suggests that segmental duplication significantly contributes to the expansion of the ZoHSP90 gene family. Ka/Ks analysis of the homologous gene pairs showed that all *ZoHSP90* genes underwent purifying selection, indicating a high degree of evolutionary conservation [33]. Duplicated genes may exhibit functional redundancy, highlighting the importance of functional studies to understand their underlying evolutionary mechanisms. All ZoHSP90 proteins are acidic, which is consistent with studies on Arabidopsis and tomato. To better explore the functions of the *HSP90* genes, we predicted the subcellular localization of the 11 *ZoHSP90* genes. It was found that the majority of HSP90 family members are located in the cytoplasm, endoplasmic reticulum, and mitochondria, which aligns with the results of Wang et al. [34].

The HSP90 gene family in plants exhibits significant conservation and diversity in terms of motifs, introns, and exon characteristics, reflecting a balance between functional conservation and environmental adaptability. In ginger, a total of 10 conserved motifs were identified. The differences in motif distribution are closely related to the phylogenetic relationships among the genes; genes that are more closely related share greater similarities in motif number and position. This highly conserved motif distribution suggests that *HSP90* genes possess significant functional commonality and evolutionary stability. Gene structure analysis further revealed significant differences in the number of exons (3 to 20 exons) among the *HSP90* genes in ginger. These differences in exon numbers may be associated with the specialization of gene expression regulation and functional differentiation. In this study, HSP90 family members were divided into four groups (A-D) based on phylogenetic analysis, providing a framework for further interspecific comparison. Further analysis revealed that *HSP90* genes within each group typically share similar conserved motifs and exon/intron structures, reflecting functional consistency within the same evolutionary branch. Research on other plants also supports the conservation and diversity of the HSP90 gene family. For example, the *HSP90* genes in *Brachypodium distachyon* and *Dendrobium officinale* exhibit highly similar motif distributions within the same subfamily [35,36]. In *Pennisetum glaucum*, the *HSP90* gene contains five conserved amino acid characteristic sequence motifs, which are key features of HSP90 family functionality [37]. Additionally, there are notable differences in the number of introns among *HSP90* genes in different plants; for instance, cytosolic *HSP90* genes in Arabidopsis have fewer introns, while organellar *HSP90* genes contain 18 or 19 introns [15]. The *HSP90* gene in *Pennisetum glaucum* consists of 3 exons and 2 introns, with the positions and phases of its introns being highly conserved among other plant cytosolic *HSP90* genes [37]. The structure of protein determines its function [38]. We found that *ZoHSP90.3*, *ZoHSP90.10* and *AtHSP90.6* formed a monophygenetic clade with high bootstrap value (100), suggesting that they have similar functions. Of particular interest is the fact that previous studies have shown that overexpression of *AtHSP90.6* enhances thermotolerance and mitigates high-temperature-induced mitochondrial damage in *Arabidopsis thaliana* [39]. However, the functional significance of these groups still needs to be further explored, such as whether they are related to specific subcellular localization (e.g., the endoplasmic reticulum or cytoplasm) or different types of stress responses. Future research may help to elucidate the biological significance of these groups by combining functional experiments and evolutionary analyses. In addition, *ZoHSP90.1* and *ZoHSP90.10* belong to the same subfamily as *AtHSP90.5*. Previous studies have demonstrated that *AtHSP90.5* plays a pivotal role in maintaining chloroplast homeostasis by modulating reactive oxygen species (ROS) levels and cooperating with antioxidant enzymes such as SOD and APX [40]. Moreover, recent studies have proposed that HSP90 may participate in membrane remodeling via phospholipid interactions, further supporting its potential function in membrane stabilization under stress conditions. Additionally, the evolutionarily conserved role of *HSP90* genes in seed development suggests that *AtHSP90.5* may contribute to maintaining embryonic viability through analogous mechanisms. Collectively, these findings underscore the multifaceted functions of *AtHSP90.5* in plant stress responses and developmental regulation [41,42,43]. In summary, the conservation of motifs, intron, and exon characteristics in the HSP90 gene family reflects its important functional status in plant genomes, while structural differences among various species may indicate its adaptability to diverse environmental pressures. The study of these features not only provides important clues for revealing the functions of *HSP90* genes in plant responses to environmental stress but also lays the groundwork for further exploration of their molecular evolutionary mechanisms and functional specialization.

**Figure 9:**
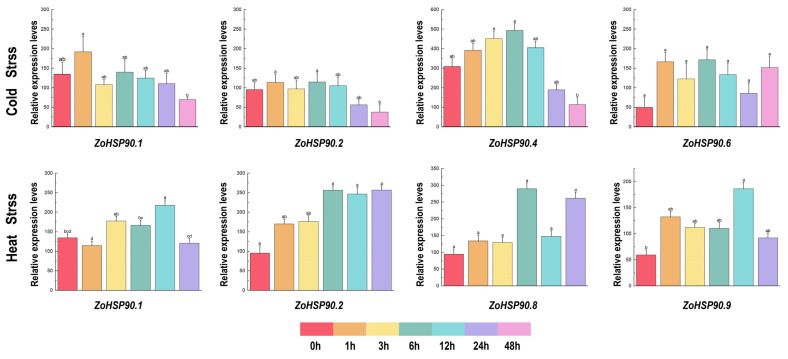
Expression of ginger *HSP90* genes under low temperature and high temperature stress. Different lowercase letters on the same column chart indicate significant differences between different treatments (*p* < 0.05).

The cis-acting elements of plant promoters determine the transcription initiation sites and transcription efficiency, regulating the expression of downstream genes. In various plants, such as pumpkins, ginkgo (*Liriodendron chinense*), and cabbage (*Brassica oleracea*), the promoter regions of the *HSP90* genes harbor cis-acting elements associated with hormone signaling pathways. These elements may participate in the plant’s response to hormonal signals, thereby regulating growth and stress responses [12,17,44]. In *Agrostis stolonifera*, AsHSP17 regulates ABA biosynthesis and ABA-independent stress signaling [45]. In Mediterranean olives (*Olea europaea*) and pumpkins (*Cucurbita moschata*), the promoter regions of the *HSP90* genes contain cis-acting elements related to light response, suggesting that these genes may be regulated under light conditions [12,14]. Furthermore, in olives, pumpkins, oats (*Avena sativa*), and cabbages, the promoter regions of the *HSP90* genes contain cis-acting elements related to various stress conditions, such as heat, cold, salt, and heavy metals. These elements play vital roles in helping plants cope with environmental stress [12,14,16]. In our study, we found that *ZoHSP90.1*, *ZoHSP90.4*, and *ZoHSP90.6* each contain multiple low-temperature response elements in their promoter regions. Consistent with this finding, we demonstrated a marked upregulation of all three genes following low-temperature treatment, with *ZoHSP90.1* exhibiting the most substantial induction (Figure 9). These results strongly suggest that these HSP90 genes may play coordinated, but distinct, roles in mediating cold adaptation responses in ginger. However, the proposed gene functions of these genes need to be validated experimentally, necessitating further investigation to guide future research.

**Figure 10:**
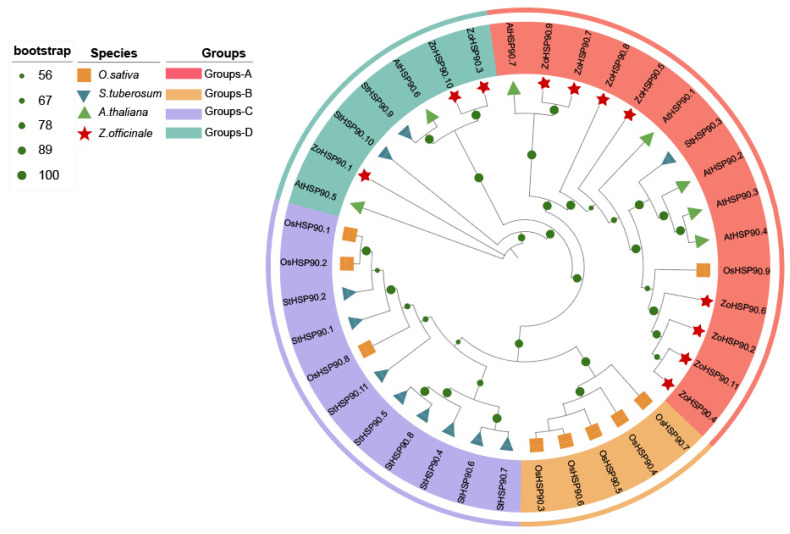
Phylogenetic tree based on the amino acid sequences of HSP90 proteins from *O*. *sativa*, *S. tuberosum*, *A*. *thaliana*, and *Z*. *officinale*. The bootstrap values are represented by the size of the green pie charts on the branches.

The HSP90 gene family plays a crucial role in plant responses to abiotic stress, and the expression patterns of these genes under different stress conditions provide important insights into their functions [46,47,48]. In ginger, the expression of the *HSP90* gene family exhibits significant tissue specificity and stress response characteristics. Analysis of RNA-Seq data revealed dynamic expression patterns of ginger *HSP90* genes under various abiotic stress conditions. In response to drought stress, *ZoHSP90.6*, *ZoHSP90.7*, and *ZoHSP90.9* exhibited high expression after levels at 3 and 12 h, whereas *ZoHSP90.5* and *ZoHSP90.8* showed almost no expression after 1 h, suggesting that these genes may be involved in early and late drought responses, respectively. Notably, our findings demonstrate that *ZoHSP90.1* and *ZoHSP90.4* display a unique dual-responsive pattern under both heat and cold stress conditions (Figure 9). The expression of *ZoHSP90.4* peaked at 6 h in low-temperature stress, and remained highly expressed after 24 h of high-temperature stress. We speculated that *ZoHSP90.1* and *ZoHSP90.4* are involved in both low-temperature and high-temperature stress responses in ginger. Phylogenetic analysis (Figure 10) reveals that *ZoHSP90.1* clusters with *AtHSP90.5* within the same evolutionary group, suggesting functional conservation among these HSP90 members. Furthermore, sequence alignment indicates *ZoHSP90.4* shares high homology with both *AtHSP90.4* and *OsHSP90.9* which has been extensively characterized in *Oryza sativa* as a critical regulator of abiotic stress responses and developmental processes [49]. While these functional insights from model plants offer critical references, determining whether *ZoHSP90.1* and *ZoHSP90.4* maintain similar molecular functions in ginger requires systematic experimental validation, particularly given the unique secondary metabolism and stress adaptation mechanisms of this medicinal crop. Under salt stress, most *HSP90* genes peaked in expression at 3 h, while *ZoHSP90.3* and *ZoHSP90.10* reached their peak at 6 h, followed by a gradual decline, indicating that these genes may have important functions in the early and mid-phase responses to salt stress. Compared to studies in other species, the expression patterns of the ginger *HSP90* gene family exhibit both conservation and specificity. For example, in maize, *ZmHsp90-1* is expressed significantly higher in roots than in stems and leaves, suggesting its important role in root growth and stress response [50,51]. In rice, *OsHSP90-2* and *OsHSP90-4* are positively regulated under drought and heat stress, indicating their significance in drought and heat stress responses [52]. Additionally, *HSP90* in Arabidopsis regulates stomatal development through interaction with the YODA signaling pathway, thereby modulating stomatal density under heat stress [53]. In perennial ryegrass and canola, *HSP90* genes have also been implicated in responses to high temperatures, salt stress, and pathogen infection [19,32].

*HSP90* genes have highly conserved functions in plant stress responses, while different species may adapt their *HSP90* genes through specific regulatory mechanisms to suit their unique growth environments. The differential expression patterns of ginger *HSP90* genes under high-temperature, low-temperature, drought, and salt stress, together with findings from other species, suggest that the HSP90 gene family in ginger has multifunctionality and dynamic regulation in stress adaptation. This study provides a foundation for further investigation into the molecular functions of ginger *HSP90* genes and their regulatory mechanisms in response to abiotic stress, while also offering potential targets for genetic improvement. These findings not only deepen our understanding of the HSP90 gene family but also offer new insights into enhancing plant adaptability to environmental stress.

## 4. Materials and Methods

### 4.1. Cultivation and Treatment of Experimental Materials

The experimental material selected for this study is the bamboo root ginger (*Zingiber officinale* cv. ‘Zhugen’), which is a well-known vegetable in Chongqing. The ginger plants were cultivated in seedling pots (50 cm × 21 cm × 25 cm) with a substrate mixture of garden soil, nutrient soil, and perlite in a 2:1:1 ratio. The experiment was conducted at the College of Smart Agriculture, Chongqing University of Arts and Sciences, with the following cultivation conditions: temperature at 25 °C, relative humidity at 75%, and a light cycle from 6:00 AM to 8:00 PM. Ten ginger plants were co-cultivated under a combination of black shade netting and LED lighting for 60 days. After this period, the plants were placed in an outdoor environment with a maximum temperature of 40 °C and a light intensity of up to 1920 µmol m^−2^ s^−1^ (Supplementary Table S1). Leaf samples were collected at 8:30 AM and 3:00 PM on the first day, and at 3:00 PM on the second, third, and fourth days, collecting 3–5 fully functional leaves for each sample with three replicates to ensure accuracy. The collected leaves were quickly frozen in liquid nitrogen for 20 min and then stored in a −80 °C freezer for subsequent analysis.

### 4.2. Identification of Ginger HSP90 Gene Family

Based on the existing ginger whole-genome protein sequence file and gene annotation file (v3.0) from the research group [23], whole-genome protein sequence files and gene annotation files for Arabidopsis, potato, and rice were downloaded from the Ensembl Plants website (http://plants.ensembl.org/index.html, accessed on 2 January 2025). The hidden Markov model (PF00183) of the HATPase (HATPase_Hsp90-like) of HSP90 was downloaded from Pfam 37.3 (http://pfam.xfam.org/, accessed on 2 January 2025) and used as the search model with an E-value threshold of <0.001. The HMMER (v3.4) subprogram, *hmmsearch* (http://www.hmmer.org/, accessed on 2 January 2025), was used to construct a local protein database and perform the search [54]. To prevent omissions, domain predictions were made using the NCBI-CDD database (https://www.ncbi.nlm.nih.gov/Structure/bwrpsb/bwrpsb.cgi, accessed on 2 January 2025) and the SMART website (http://smart.embl-heidelberg.de/, accessed on 2 January 2025) to eliminate sequences lacking HSP90 family protein domains [55,56]. The physicochemical properties of the ginger HSP90 gene family were analyzed using the online ExPASy-Compute pI/Mw tool (https://web.expasy.org/compute_pi/, accessed on 5 January 2025) [57], and the subcellular localization of HSP90 family members was predicted online using the WoLF PSORT website (https://wolfpsort.hgc.jp/, accessed on 8 January 2025).

### 4.3. Collinearity, Phylogenetic Analysis and Protein Interaction Network Prediction of the Ginger HSP90 Gene Family

Gene sequence and annotation files for *Arabidopsis thaliana*, *Musa acuminata*, *Zingiber officinale* (ginger), and *Oryza sativa* (rice) were downloaded from NCBI. Gene homology was identified using the One Step MCScanX in TBtools (v2.225) [58], and the results were visualized using Circos. The amino acid sequences of rice, Arabidopsis, potato, and ginger were aligned using ClustalW, and a phylogenetic tree for multiple species was constructed using IQ-Tree with the maximum likelihood (ML) method (bootstrap value = 1000; best models: LG + G) [59]. The phylogenetic tree was visualized using iTOL (v7.2).

### 4.4. Analysis of Conserved Motifs, Gene Structure, and Cis-Acting Elements of Ginger HSP90

Conserved motifs of the ginger HSP90 gene were analyzed using the online tool MEME Suite (http://meme-suite.org/) with the following parameters: an E-value threshold of 10.0, searching for 10 motifs with widths ranging from 6 to 50 nucleotides, and the zoops (zero or one occurrence per sequence) mode [60]. The gene structure of *HSP90* was visualized using TBtools (v2.225) [61]. The first 2000 bp of the promoter region of the ginger HSP90 gene were extracted from the gene file using TBtools (v2.225) and submitted to PlantCARE (https://bioinformatics.psb.ugent.be/webtools/plantcare/html/, accessed on 13 January 2025) for analysis [62]. The results from the analysis were sorted, filtered, and visualized using TBtools (v2.225).

### 4.5. Expression Analysis of Ginger HSP90 Gene in Response to Stress

We used 60-day-old ginger seedlings as experimental materials and subjected them to four types of abiotic stress treatments: low-temperature, drought, high-temperature [63], and salt stress [64]. Each treatment group consisted of 10 ginger seedlings, placed in a temperature-controlled incubator, with temperatures set at 4 °C for low-temperature stress and 40 °C for high-temperature stress. For drought stress treatment, the ginger roots were immersed in a 15% PEG6000 culture medium. Additionally, the roots of the ginger plants were immersed in a 200 mM NaCl solution for the salt stress experiment. Leaf samples (the 3rd to 5th fully functional leaves from the top) were collected at 0, 1, 3, 6, 12, 24, and 48 h for low temperature, drought, and salt stress treatments. The samples were quickly frozen in liquid nitrogen and stored in a −80 °C freezer for subsequent analysis. Similarly, leaf samples for the high-temperature stress treatment were collected at 0, 1, 3, 6, 12, and 24 h, with three replicates for each treatment.

The sequencing-derived transcriptome data were visualized using the HeatMap tool in TBtools (v2.225). Total RNA was extracted from the four stress treatments using the TRIzol reagent (Invitrogen, Waltham, CA, USA). cDNA was synthesized according to the manufacturer’s instructions using the MonScript™ RTIII ALL-in-mixed dsDNase kit (Monad, Suzhou, China). Primers for the *ZoHSP90* gene were designed using Primer (Supplementary Table S2), and qRT-PCR was performed to analyze the response of *ZoHSP90* to high temperature and strong light stress. Gene expression data were normalized relative to the stably expressed *ZoTUB2* reference gene (*ZOFF_005593*), and quantitative real-time PCR analyses were conducted in technical triplicates using a CFX96 system (Bio-Rad). Thermal cycling was initiated with a 95 °C denaturation step for 30 s, followed by 40 cycles of 95 °C for 10 s and 60 °C for 30 s. To ensure the biological reliability of the results, each reaction was performed in triplicate as technical replicates. Gene expression was quantified using the 2^−∆∆Ct^ method.

### 4.6. Statistical Analyses

We used Microsoft Excel 2016 to organize data, SPSS (IBM SPSS Statistics 27.0.1) software to perform data analysis under Duncan’s test (*p* < 0.05), Origin (Origin 2021) and AI (Adobe Illustrator 2021) for plotting.

## 5. Conclusions

In conclusion, this study highlights the significant role of the HSP90 gene family in enhancing the resilience of ginger to various abiotic stresses, including high temperatures, drought, and salinity. The identification of 11 *HSP90* genes in ginger, the highest number reported in any studied plant species to date, underscores the evolutionary significance and functional diversity of this gene family. The tissue-specific expression patterns observed, along with dynamic responses to environmental stresses, suggest that different *HSP90* genes may have specialized roles in the plant’s development and stress adaptation. The conservation of motifs and the variation in intron and exon structures among ginger *HSP90* genes indicate a balance between functional conservation and environmental adaptability, reflecting the evolutionary pressures on plants. Our comparative genomic and synteny analyses revealed evolutionary conservation of *HSP90* genes across diverse plant lineages. Notably, we identified significant sequence homology between ginger and Arabidopsis *HSP90* genes, suggesting remarkable functional conservation of this molecular chaperone family, even between these evolutionarily distant species. Furthermore, we detected a precise one-to-one orthologous relationship between a single *HSP90* gene in rice and its ginger counterpart, indicating strong purifying selection has maintained this particular gene copy throughout monocot evolution. Collectively, these findings demonstrate both deep conservation across angiosperms and the evolutionary stability of *HSP90* genes.

Furthermore, the presence of various cis-acting elements in the promoter regions of these genes indicates their involvement in hormone signaling and stress responses pathways, further supporting the role of *HSP90* genes as critical regulators in plant adaptation to environmental changes. Overall, the findings from this research not only deepen our understanding of the molecular functions of the HSP90 gene family in *Zingiber officinale*, but also provide valuable implications for breeding and biotechnological strategies aimed at enhancing crop resilience to the challenges posed by environmental change. Future studies should focus on elucidating the specific regulatory mechanisms and functional roles of individual *HSP90* genes, laying the groundwork for innovative strategies aimed at enhancing agricultural productivity and sustainability in the face of increasing environmental stresses.

## Figures and Tables

**Table 1:** Physicochemical Properties Analysis of the Ginger *HSP90* Gene Family Members.

Gene Name	Gene ID	Chromosome	Length	Mw (KDa)	PI	Instability Index	Subcellular Localization (Prediction)
*ZoHSP90.1*	Maker00069539	Chr.08	798	38.71	6.72	78.45	Endoplasmic reticulum
*ZoHSP90.2*	Maker00025278	Chr.10	306	25.82	9.16	82.29	Cytoplasm
*ZoHSP90.3*	Maker00026777	Chr.10	799	49.34	9.38	78.96	Endoplasmic reticulum
*ZoHSP90.4*	Maker00053883	Chr.10	306	61.24	6.73	82.61	Cytoplasm
*ZoHSP90.5*	Maker00076528	Chr.10	330	48.45	7.00	84.85	Endoplasmic reticulum
*ZoHSP90.6*	Maker00064857	Chr.16	325	39.81	8.47	81.97	Cytoplasm
*ZoHSP90.7*	Maker00070892	Chr.16	886	54.58	6.68	84.28	Endoplasmic reticulum/Mitochondrion
*ZoHSP90.8*	Maker00030538	Chr.18	332	66.94	8.24	86.39	Cytoplasm
*ZoHSP90.9*	Maker00057709	Chr.18	736	30.89	5.77	82	Endoplasmic reticulum/Mitochondrion
*ZoHSP90.10*	Maker00052350	Chr.22	802	32.45	6.01	79.61	Endoplasmic reticulum
*ZoHSP90.11*	Maker00052464	Chr.22	327	33.88	7.21	85.02	Cytoplasm

**Table 2:** Duplicated *HSP90* genes in ginger. S-sites refer to the number of synonymous sites; N-sites refer to the number of non-synonymous sites; Ka is the non-synonymous substitution rate; Ks is the synonymous substitution rate; and Mya denotes million years.

Paralog Pairs	S-Sites	N-Sites	Ka	Ks	Ka/Ks	Duplication Time (Mya)
*ZoHSP90.7-ZoHSP90.9*	463.92	1696.08	0.06	0.59	0.10	9.9
*ZoHSP90.6-ZoHSP90.4*	202.92	715.08	0.02	0.64	0.03	10.7
*ZoHSP90.6-ZoHSP90.2*	201.58	716.42	0.02	1.02	0.02	17.0
*ZoHSP90.11-ZoHSP90.2*	201.00	717.10	0.01	0.69	0.02	11.6
*ZoHSP90.10-ZoHSP90.3*	528.75	1868.25	0.04	0.41	0.11	6.8

## Data Availability

The relevant datasets used and/or analyzed during the current study can be made available upon request from the corresponding author.

## Supplementary Materials

The following supporting information can be downloaded at https://www.mdpi.com/article/10.3390/plants14111660/s1: Supplementary Table S1: Cultivation conditions under natural high temperature and strong light; Supplementary Table S2: The primer sequences for qRT-PCR used in this study.
